# Editorial: The HSP70 Molecular Chaperone Machines

**DOI:** 10.3389/fmolb.2017.00001

**Published:** 2017-01-24

**Authors:** Pierre Goloubinoff

**Affiliations:** Department of Plant Molecular Biology, Faculty of Biology and Medicine, University of LausanneLausanne, Switzerland

**Keywords:** unfoldase, disaggregase, HSC70, J- domain cochaperones, nucleotide exchanges factors, Hsp110s, protein misfolding, protein aggregation

The HSP70s belong to a small family of highly conserved ~70 kDa enzymes that can use the energy of ATP-hydrolysis to modify the structure, and consequently the function, of specific native proteins, and to unfold, solubilize, and thereby reduce the cellular concentration of harmful misfolded proteins (Finka et al., 2016). Particular HSP70s are expressed constitutively in the cytosol of bacteria and in all the ATP-containing compartments of eukaryotic cells. In unstressed bacteria, plant, and animal cells, HSP70s are 0.5–2% of the total protein mass (Finka and Goloubinoff, 2013). They form the central hub of the chaperone network that controls all aspects of cellular protein homeostasis: protein *de novo* synthesis, protein translocation across membranes, native folding, and oligomer assembly. HSP70s also participate in the active removal of toxic protein aggregates by actively converting them into harmless degraded peptides, or into natively refolded functional proteins (Calamini and Morimoto, 2012; Finka et al., 2016).

In immortalized human cancer cells and in naïve rat livers cells for example, HSPA8 is the major HSP70 species that is constitutively expressed in the cytosol, accounting for about half of the total mass of the HSP70s (Finka and Goloubinoff, 2013). In heat-shocked cells, particular HSP70s accumulate. Hence, following a 4 h mild fever-like heat-shock at 41°C, the total mass of the HSP70s in Jurkat cells increases 1.6-folds, from ~0.7% (at 37°C) to ~1.1% (Finka et al., 2015). Thus, although sharing 90% sequence identity with HSPA8, the cytosolic HSPA1A, which generally remains undetected in unstressed tissues, strongly accumulates during various abiotic stresses (Finka et al., 2015). Noticeably, non-heat-shocked cancer cells generally express constitutively abnormally elevated levels of HSPA1A that may even exceed the naturally high amounts of HSPA8. High expression levels of HSPA1A in otherwise unstressed tissues is thus a hallmark of malignancy and of poor survival outcome (Feder et al., 1992; Finka and Goloubinoff, 2013; Yang et al., 2015).

The *endoplasmic reticulum* HSP70, HSPA5 (called BIP), and the mitochondrial HSP70, HSPA9 (called mortalin) are the next most abundant HSP70 species. As in the case of cytosolic HSPA8, their concentration may also increase in response to heat-shock (Finka et al., 2015). In addition to their function in protein quality control, they act as pulling motors that import cytosolic polypeptides into their respective organelles.

Assisted by over 30 different J-domain cochaperones in eukaryotes (Dekker et al.) and various nucleotide exchange factors (NEFs) (Bracher and Verghese), the HSP70s can control a great number of housekeeping cellular processes. They can use the energy of ATP-hydrolysis to convert active (alter)native protein complexes into differently active native polypeptides. Thus, at the exit of ribosomes, specific HSP70s can pull and regulate the *de-novo* folding of nascent polypeptides. Others can pull and unfold cytosolic polypeptides necessary for their translocation across membranes into organelles. Yet other HSPAs can regulate the post-translocation folding and assembly of (alter)natively folded protein complexes and coordinate their timely disassembly into differently active sub-complexes, as in the case of clathrin cages and triskalions (Sousa and Lafer; De Los Rios and Goloubinoff, 2016). HSP70s can also determine the lifespan of native cellular proteins by timely targeting them to chaperone-gated proteases, as in the case of the bacterial transcription factor σ32, which is targeted by DnaK to the FtsH protease (Rodriguez et al., 2008; VanPelt and Page, 2017). HSP70s also target misfolded proteins to the lysosome in a process called chaperone-mediated-autophagy (Cuervo and Wong, 2014).

Some HSP70s can act as ATP-fuelled molecular motors that, together with specific cochaperones, such as their eukaryotic HSP110 cognates (Mattoo et al., 2013; Nillegoda and Bukau) or the ClpB/HSP104s, which are non-cognate AAA+ ATPases (Mogk et al., 1999), can solubilize stable aggregates and convert them into harmless natively refolded or degraded polypeptides. Thus, together, the HSP70s form a powerful cellular defense network against harmful misfolded proteins. HSP70s failure to be expressed, or to function in aging metazoan cells, directly correlates with the late age onset of severe human degenerative diseases, such as Parkinson, Alzheimer, and type 2 diabetes that are associated to the accumulation of misfolded proteins in the damaged tissues (Hartl, 2016).

In this research topic, several reviews tackled from different complementing angles the central question: how may the members of a small class of relatively simple two-domain 70 kDa molecules use ATP to drive dramatic conformational changes, in so diverse aggregated, misfolded, and alternatively folded polypeptides, and thus control such a great number of different housekeeping and stress related biological functions in the cell?

In their review “Insight into the molecular mechanism of allostery in HSP70s,” Mayer and Kityk described the intricate molecular paths of various allosteric signals during the HSP70 ATPase cycle, leading to dramatic movements of the two subdomains that bind the polypeptide substrates. They focused on specific residues that are involved in various key allosteric signals in the chaperone molecule, such as the signal that is issued by the substrate upon binding to the base of the substrate binding domain (SBD) to induce ATP hydrolysis in the nucleotide binding domain (NBD). Another signal is issued by the NBD to induce the closure of the SBD's lid onto its base, and yet another signal that is issued by the NEF-triggered NBD to induce the sequential release of the ADP from the NBD and of the structurally modified polypeptide from the SBD.

In their review “Multi-layered molecular mechanisms of polypeptide holding, unfolding and disaggregation by HSP70/HSP110chaperones” Finka et al. addressed the apparent consequences of the coordinated subdomain movements in the HSP70 molecule during the ATPase cycle, on the bound misfolded or (alter)natively folded polypeptide substrates, leading to their conversion into native or degraded polypeptides. Two complementing mechanisms are being discussed: (1) unfolding by direct clamping, in which the closure of the SBD lid onto a bulky misfolded protein segment and its consequent squeezing against the SBD base would result in the local unfolding of the bound misfolded segment. (2) Unfolding by entropic pulling, in which the distal binding of several HSP70 molecules onto the same misfolded polypeptide, and their ensuing independent dangling motions would generate a force capable of unfolding by entropic pulling the misfolded segments that are in between two HSP70-bound sites (Goloubinoff and De Los Rios, 2007).

The various allosteric paths leading to sub-domain movements and their possible effects on a bound misfolded substrate can be summarized as follow: In an ATP-bound HSP70, there is a tight crosstalk between a J-domain from a DNAJ co-chaperone anchored in the NBD, and a hydrophobic misfolded polypeptide substrate newly bound to the wide-open SBD. Both then concomitantly trigger ATP hydrolysis in the NBD and the closing of the SBD's lid that applies a local pressure onto a bulky misfolded segment in the bound substrate leading, upon tight engulfment by the SBD, to the extensive unfolding of the substrate (Sharma et al., 2010; Mashaghi et al., 2016). The subsequent transient binding of a NEF to the distal lobes of the NBD then triggers ADP release from the NBD and the opening of the protein-binding lid, allowing the newly unfolded polypeptide to dissociate and spontaneously refold to the native state.

However effective an unfolding nanomachine may the HSP70 molecule be, it remains quiet ineffective at choosing its substrates among the many different polypeptides in need to be structurally modified. Substrate discrimination by the HSP70 system is predominantly achieved by the J-domain co-chaperones that initially interact with the different substrates and at the same time bind the HSP70s through their J-domain.

In their review “DNAJs: more than substrate delivery to HSPA,” Dekker et al. highlighted the crucial role of J-domain cochaperones to the protein quality control of metazoan cells. DNAJs can target HSP70s onto misfolded, aggregated, or specific alternatively folded protein substrates, to be unfolded, refolded, or ultimately degraded by chaperone-gated proteases. In addition, specific DNAJs are involved in the unidirectional translocation of cytosolic pre-proteins across membranes to the ER, the mitochondria, the lysosome, and in plants also the chloroplast and the peroxisome. Other DNAJs carry unique anti-aggregation functions of their own, as in the case of mammalian DNAJB6, which is a very potent anti-aggregation chaperone that prevents the formation of toxic polyglutamine species that cause severe degenerative diseases, such as Huntington's chorea.

In their review, “Metazoan HSP70-based protein disaggregases: emergence and mechanisms,” Nillegoda and Bukau further dwelt on the key role of J-domain cochaperones of HSP70, in particular the human DNAJA2 and DNAJB1, which seem to specifically recruit HSC70 and its sequence-related HSP110 co-chaperone, to form powerful disaggregating hetero-oligomers capable of solubilizing denatured reporter proteins, among them, stable alfa-synuclein fibrils (Gao et al., 2015; Finka et al., 2016). Two putative “clamp and walk” and “metazoan nucleation” models are discussed for the HSP70- and HSP110-mediated disaggregation mechanism.

In contrast to misfolded protein aggregates serving as substrates to the HSC70-HSP110 disaggregation machinery, in their review “The role of molecular chaperones in clathrin mediated vesicular trafficking,” Sousa and Lafer discuss the role of a specific J-domain cochaperone, auxilin, in the recruitment of HSC70 and HSP110 to a specific position onto a natively assembled protein complex: the clathrin cage. There, HSC70 uses ATP to pull and disassemble clathrin coats by a “steric wedge” mechanism, which is very similar to entropic pulling. Recently, the same authors brought evidence that loose HSC70 multimers might form and thus join their “steric wedge” entropic pulling forces to disassemble clathrin cages more efficiency. HSC70 also sequesters the depolymerized clathrin, implying that triskelion release must be regulated by HSP110 (Sousa et al., 2016). Interestingly, in neurons, HSP110 activity as a specific NEF for HSC70 is regulated upon phosphorylation by a Calcium-regulated kinase (Ishihara et al., 2003). Thus, following the vesicle fusion with the plasma membrane and the discharge of their neuro-transmitters content in the synapse, the transient entry of Ca^2+^ ions in the cytosol would be able to precisely signal the onset of clathrin cage dismantling for triskelion recycling.

In their review “The nucleotide exchange factors of HSP70 molecular chaperones” (Bracher and Verghese), Bracher and Verghese discussed the large diversity of the eukaryotic NEFs. In addition to the HSP110s, which are sequence wise-, structure wise-, and function wise-related to the HSP70s (Mattoo et al., 2013), there are unrelated HSPBP1/Sil1, BAG domain proteins and the M-domain of the HSP104 disaggregase cochaperone (Carroni et al., 2014; Doyle et al., 2015), and the GrpE type of NEFs to be found in prokaryotes and eukaryotic organelles of eubacteria origin. Despite their great disparity of structure, the various NEFs, interact with the same surfaces on the HSP70s: the tips of the two NBD subdomains embracing the ADP-binding pocket.

It should be noted that BAG domain proteins and GrpE do not have nucleotide binding sites. Moreover, both GrpE and HSP110 were shown to induce polypeptide release, respectively, from DnaK and HSP70, in the total absence of ATP (Sharma et al., 2010; Mattoo et al., 2013). Thus, these versatile regulatory co-chaperones should be called ADP- and substrate-release factors rather than, nucleotide exchange factors.

Within cells, HSP70s can use ATP to drive structural changes in other cellular proteins. Yet, extracellular HSP70 is also to be found at the surface of cells facing the ATP-less outward milieu. In mammals extracellular HSP70 can stimulate innate immune responses and facilitate the cross-presentation of immunogenic peptides, in association with major histocompatibility complex (MHC) antigens (Shevtsov and Multhoff, 2016).

Providing biological context to this research topic, Przyborski et al. (Bracher and Verghese) discussed in their review “Plasmodial HSP70s are functionally adapted to the malaria parasite life cycle” the role HSP70 in a severe human disease: malaria. The parasite *Plasmodium falciparum* encodes for half a dozen different HSP70s, which are key to the survival and the pathology of the parasite in its various stages of its development, in particular for its resistance to high temperatures episodes during human fever, as well as of low ambient temperature when dwelling in the salivary glands of the mosquitos.

In conclusion, whereas the lack of an appropriate level of HSP70 expression in metazoan neural and muscle tissues, without stress and following biotic and abiotic stresses, has been associated to the onset of excessive damaging apoptosis, leading to degenerative tissue loss and accelerated aging (Kimura et al., 2007; Ben-Zvi et al., 2009; Morimoto and Cuervo, 2014), excessive accumulation of HSP70s has been linked to therapeutic resistance of carcinomas, increased metastasis, and poor survival outcomes to anti-cancer therapies (Sherman and Gabai, 2015; Figure 1). Yet, compared to terminally differentiated cells, rapidly growing embryonic cells that are not cancerous, also express high HSP70 and HSP90 levels (Jensen et al., 2013). Thus, the effectiveness of the various HSP70s at maintaining cellular protein homeostasis and controlling life span likely depends on yet to be characterized, fine-tuned qualitative differences in the expression and activation levels of the various HSP70s and their many J-domain, NEF, and disaggregase cochaperones.

## Author contributions

The manuscript was written solely by PG.

## Funding

Research is supported by Grant 31003A_156948 from the Swiss National Fund.

### Conflict of interest statement

The author declares that the research was conducted in the absence of any commercial or financial relationships that could be construed as a potential conflict of interest.

## Abbreviations

SBD: Substrate binding domain
NBD: Nucleotide binding domain
NEF: Nucleotide Exchange Factor
Natively folded proteins: proteins folded in a functional native conformation
(Alter)natively folded proteins: proteins folded in a different, albeit still native conformation.
